# Intradural Melanotic Schwannoma of the Sacral Spine: An Illustrated Case Report of Diagnostic Conundrum

**DOI:** 10.3390/reports7030056

**Published:** 2024-07-16

**Authors:** Jiunn-Kai Chong, Navneet Kumar Dubey, Wen-Cheng Lo

**Affiliations:** 1 Department of Neurosurgery, Taipei Medical University Hospital, Taipei 11031, Taiwan; b9902042@gmail.com; 2 Executive Programme in Healthcare Management, Indian Institute of Management, Lucknow 226013, India; bioengineer.nkd@gmail.com; 3 Division of Neurosurgery, Department of Surgery, School of Medicine, College of Medicine, Taipei Medical University, Taipei 11031, Taiwan; 4 Taipei Neuroscience Institute, Taipei Medical University, Taipei 11031, Taiwan

**Keywords:** intradural melanotic schwannoma, melanin, laminectomy

## Abstract

Schwannomas are benign and slow-growing peripheral nerve sheath neoplasms of Schwann cells. These are generally encountered in the neck, head, and flexor areas of the extremities. Even though many schwannomas are easily diagnosable, their variable morphology can occasionally create difficulty in diagnosis. In this study, we present a rare case of melanotic schwannoma of the sacrum, emphasizing the need for routine biopsy to understand the etiology. A 46-year-old man presented to the Department of Neurosurgery, Taipei Medical University Hospital, with buttock pain in the sacrum area for 1 year, which worsened in the last 1–2 months. The patient had no known history of trauma or malignancy. We evidenced an intradural extramedullary neurogenic tumor at the caudal end from S1 to S3. Histologic analysis revealed melanin deposition in the tumor cells. Round to oval tumor cells were positive for HMB-45 and S-100 proteins, suggestive of melanotic Schwannoma, which were removed by laminectomy. After 1 month, the tumor recurred and was further removed surgically. Conclusively, we observed the sacrum as an unusual anatomic site for the possible occurrence of melanotic schwannoma, especially in patients with no known history of trauma and malignancy. The possibility of melanotic schwannoma is very high. We hypothesize that melanotic schwannoma was possible because it occurred in the intradural and extramedullary regions of the spine. Hence, a routine biopsy should be performed to corroborate the exact cause and prevent incorrect presumptions.

## 1. Introduction

Spinal schwannomas are slow-growing tumors that originate from Schwann cells, forming a protective layer around nerve fibers in the peripheral nervous system [1]. Schwannomas contain melanosomes based on their maturation stages [2]. Reportedly, melanotic schwannoma cells possess the capacity for melanogenesis [3], and it has been proposed that neoplastic Schwann cells could undergo melanomatous transformation [4]. Reportedly, these tumors are typically observed in individuals between the ages of 40 and 50. According to Bendszus et al., MRI characteristics of melanotic schwannoma have been described as hyperintense lesions on T1-weighted images and hypointense on T2-weighted images [5]. Although extensive reviews focusing on the imaging of melanotic schwannoma are lacking, it is widely accepted that melanotic schwannoma typically exhibits hyperintensity on T1-weighted sequences and hypointensity on T2-weighted sequences. Clinically, several studies have noted the malignant and metastatic potential of melanotic schwannoma, but the outcomes have been heterogeneous [6,7].

In this case report, we describe an intradural melanotic schwannoma of a 46-year-old man.

## 2. Detailed Case Presentation

**Figure 1:**
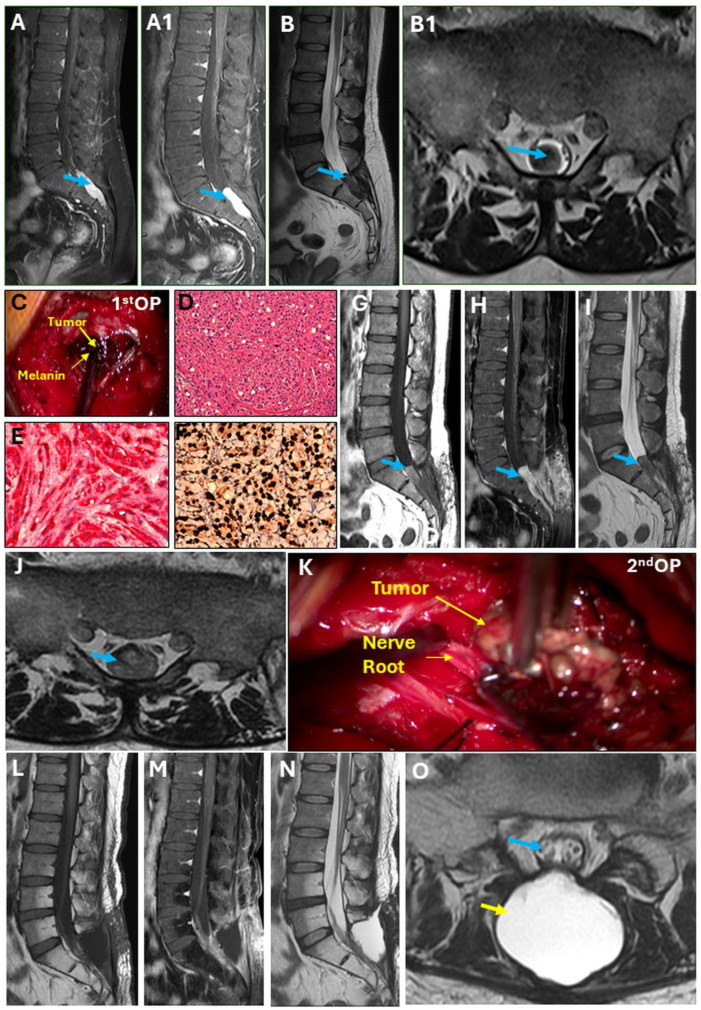
Intradural melanotic schwannoma. (**A**) Sagittal view showing sacral lesion at the S1–S3 with hyper-intensity on T1-weighted imaging (T1WI); heterogenous. (**A1**) The sagittal view revealed a sacral lesion with intense contrast enhancement (arrow), which was homogenous. (**B**) Sagittal view on T2WI axial MRI showing hypotense tumor infiltration into the spinal canal (**B1**). (**C**) Tumor with a bluish-black appearance during first surgery. (**D**) Hematoxylin and eosin stain, 200× magnification. Immunohistochemical stains reveal neoplastic cells positive for (**E**) HMB45, 400× magnification, and (**F**) S100, 400× magnification. (**G**,**H**) Follow-up MRI after the first surgery. (**I**) The sagittal view on T1WI showed tumor recurrence. (**J**) Follow-up MRI after the first surgery. The spinal canal was fully occupied by the tumor at the S1–S2 junction. (**K**) Tumor with a lesser bluish-black appearance than the first surgery. (**L**–**N**) Follow-up MRI after 2nd surgery showed no tumor within the spinal canal. (**O**) Post-operatively, spinal nerve within the thecal sac (blue arrow) can be seen clearly and pseu-do-meningocele (yellow arrow) was managed conservatively, which was confirmed by the absence of any symptoms.

A 46-year-old man presented to the Department of Neurosurgery, Taipei Medical University Hospital, in November 2023 with buttock pain in the sacrum area for 1 year, which worsened in the last 1–2 months [visual analog score (VAS): 7]. The exacerbating factors were sitting or lying flat. The patient’s body weight, height (cm), body mass index, pulse rate/min, and blood pressure (mmHg) were 74.3, 170 cm, 25.71, 74, and 134/80, respectively. The patient’s history showed no trauma, poor appetite, or weight loss. Regarding hypoactive sexual desire disorder, erection and ejaculation were normal. We evidenced a sacral lesion with intense contrast enhancement (arrow) at the caudal end from S1 to S3 (Figure 1(A,A1)). The sagittal view on T2-weighted axial MRI (Figure 1B) showed hypotense tumor infiltration into the spinal canal (Figure 1(B1)). The patient had been receiving conservative treatment, including activity modifications, analgesics, and muscle relaxants for 6 months, but in vain. Under the impression of an intradural neurogenic tumor at the caudal end from S1 to S3, the patient was admitted for further evaluation and management. After that, we employed the laminectomy technique to remove tumors from S1 to S3 vertebra in November 2023 (Figure 1C), which showed a bluish-black appearance. Later, the tumor sample was sent for histopathologic examinations. The hematoxylin and eosin (H&E) staining revealed infiltration of pleomorphic tumor cells in a sheet-like distribution (Figure 1D). The tumor cells were observed to have irregular hyperchromatic nuclei and a considerable amount of eosinophilic cytoplasm. Melanin deposition in the tumor cells was also observed. Tumor cells were immunoreactive to human melanoma black 45 (HMB)-45 (Figure 1E) and S100 proteins (Figure 1F). The hospital stay for the first surgery was 5 days.

In the follow-up MRI one month after the first surgery, the sagittal view on T1WI with contrast revealed tumor recurrence (Figure 1G–I), which fully occupied the spinal canal at the S1–S2 junction (Figure 1J). In December 2023, the tumor was again resected and revealed a less bluish-black appearance (Figure 1K) than during the first surgery; however, the H&E staining showed similar morphology with an increased degree of nuclear pleomorphism, larger nucleoli, and necrosis. The total time of hospital stay during the second surgery was 8 days. Follow-up MRI was performed immediately and 3 months after the second surgery (Figure 1L–N), which demonstrated no tumor within the spinal canal with a clear spinal nerve within the thecal sac (blue arrow) (Figure 1O). Post-operatively, the pseudo-meningocele (yellow arrow) was managed conservatively, as indicated by the absence of any symptoms.

### Surgical Technique

Under general anesthesia, the patient was put in a prone position. The surgical site was cleansed and draped in a sterile fashion. A midline incision was made over the sacral spine, typically over the vertebrae where the tumor was located. The skin and subcutaneous tissue were carefully dissected to expose the underlying muscles, which were further gently retracted to expose the laminae, the bony arches covering the spinal canal. The bony arches covering the spinal canal were removed using high-speed drill and bone rongeurs. After the spinal canal was adequately exposed, thecal sac was opened to identify the tumor. Careful dissection and tumor removal were performed, separating it from the surrounding nerves, blood vessels, and other structures. The tumor was meticulously dissected and removed. After confirming hemostasis, the wound was irrigated with a sterile saline solution, and the dura was closed with artificial dura.

The muscles were repositioned over the spinal column, and the incision was closed in layers with sutures. A sterile dressing was applied. The pain was much relieved after the procedure. The post-operative healing was uneventful, and he was smoothly discharged a couple of days after the surgery. However, the tumor recurred after a month and was resected. Notably, we achieved gross total resection (GTR) in both surgeries.

## 3. Discussion

Melanotic schwannoma is sporadic, and its pathogenesis is poorly understood. To date, the reported rare locations include the cerebellum, orbit, heart, trachea, bronchus, cervix, bone, soft tissue, skin [8], stomach [9], and colon [10]. As evidenced in our case, unlike common schwannomas, melanotic schwannoma is susceptible to local recurrence [11]. Melanotic schwannoma is a melanin-producing schwannoma, comprising ultrastructural characteristics of Schwann cells. Melanotic schwannoma of the sacral region is a rare schwannoma variant, composed of melanin-producing cells with ultrastructural features of Schwann cells. These tumors may be typically asymptomatic and are often detected incidentally during imaging studies for other medical conditions. As per a previous case on the thoracic spine [12], melanotic schwannoma was typically present as extramedullary masses and may have appeared like malignant melanoma; however, specific pathological biomarkers could differentiate it. Similar to ours, in another case report on intramedullary melanotic schwannoma of the cervical spine, the immunocytochemistry of the tumor sample was positive for HMB-45 and S-100 [13]. To our knowledge, no intradural melanotic schwannoma of the sacral spine has been reported. However, only one case of intraosseous melanotic schwannoma in the sacrum has been evidenced [14].

Melanotic schwannoma is generally benign; however, malignant variants can metastasize. Reportedly, 15% to 35% local recurrence and 26% to 44% metastatic spread cases have been documented within five years [6,11,15]. Recurrences typically result from tumor invasion into adjacent tissues, making complete resections challenging. However, recurrences are less frequent when melanotic schwannoma occurs in soft tissue, where complete resection is more easily achieved. In our report, we also encountered tumor recurrence after 1 month, which was surgically resected.

Hence, a histologic melanotic schwannoma diagnosis needs meticulous differential diagnostic considerations. It is worth noting that distinguishing this tumor from malignant melanoma is vital for efficacious management and planning. The primary goal of the surgery was to achieve maximal safe resection and relieve neurological symptoms without causing new neurological deficits. The patient showed remarkable clinical improvement after the first surgery, but the pain recurred after 1 month, and the follow-up image showed tumor recurrence. Therefore, we conducted a second surgery, and the patient’s symptoms completely subsided, even after 5 months of follow-up post-second surgery, which was confirmed by MRI showing no tumor recurrence. Notably, a gross total resection (GTR) was achieved in both surgeries. Unfortunately, we did not have an immediate post-op MRI because of the remarkable pain relief right after the surgery, which implies that sufficient decompression was achieved within the spinal canal. Additionally, since the patient became pain-free after the surgery and the pain recurred after 1 month, these clinical manifestations are more indicative of recurrence rather than residual effects. Therefore, an appropriate long-term follow-up is needed for this type of melanotic schwannoma. In line with our study, previous studies have reported immunopositivity of the HMB-45 [13] and S100 [11] proteins of melanotic schwannoma. In this rare case, pathology reports showed malignant melanoma; however, in our clinical opinion, melanotic schwannoma is possible as it occurred in the intradural and extramedullary region of the spine. Hence, it is less likely to be malignant melanoma. Melanoma is a primary or secondary type of primary melanoma that mainly occurs on the skin and eyes. Secondary is metastatic but rarely found in the spine. Therefore, a dilemma still exists regarding whether it is primary or secondary, and further investigation is needed. However, a close clinical long-term follow-up of such patients is highly desirable.

## 4. Conclusions

This case highlights the feasibility of rare melanotic schwannoma in the sacrum, especially in patients with no known history of trauma and malignancy. Though the exact etiology of this type of schwannoma is unclear, in our clinical opinion, melanotic schwannoma is possible as it occurred in the intradural and extramedullary region of the spine, which could be corroborated through routine biopsy with morphologic and histologic studies, as well as at genetic level to avoid further complications like tumor recurrence and metastasis.

## Data Availability

The data may be made available from the corresponding author upon reasonable request.
